# Academy of Medicine

**Published:** 1881-01

**Authors:** 


### ACADEMY OF MEDICINE.

*Functional Diseases of the Liver.*—Dr. Cleveland read a paper upon some of the supposed functional troubles of the liver. He acknowledged the difficulty of treating this subject from the fact that we do not know what the exact functions of the liver are. A resume was first given of the physiological theories that have obtained from Galen down to the present. Galen looked upon the liver as the focus of animal heat, and as the organ intended for the formation of blood, which during the process separated as waste matter yellow and black bile. These opinions were supreme in the medical world until the seventeenth century, when the discovery of the lacteal and the thoracic duct showed a way by which the chyle could get into the circulation independent of the liver. From this period up to 1820, the liver was limited in function to the secretion of bile. Later it was shown by experimental physiologists that the portal veins through the liver disposed of nutritive material as well as the lacteals, but how it is utilized is not determined. Bernard holds that it is very important factor in the production of body heat. The views of Galen, after remaining buried for two hundred years, have revived again, though modified and circumscribed. The relation that the bile sustains to digestion was discussed; the bile assists in emulsifying and digesting fats, though the exact part that it plays in digestion is not known, except that it is a necessary agent in complete and perfect digestion. The essayist also considered the influence of excessive and injudicious eating and drinking in producing congestion of the liver, and how these congestions by continued aggravations may produce organic disease of the liver. The word "bilious" was also discussed; although indeterminate, we cannot yet afford to discard it. Gastro-duodenal catarrh does not express what we mean by "biliousness," and even though it did, it is only substituting one vague term for one equally as indefinite. Our want of exact knowledge in regard to the functions of the liver necessitate the use of indefinite terms

Dr. Whittaker said that still a great deal of theory is connected with the function of the bile. All we know is that it is partly secrementitious and partly excrementitious. Legg says that since the time of Galen, nothing new in the function of the bile has been discovered. In the same manner the precise meaning of the term biliousness is not really understood. Most authors agree that it is gastro-intestinal derangement, the most common form of which is a catarrh depending on a mechanical obstruction of the bile ducts. Mercury was formerly supposed to have a specific action in promoting the biliary secretion, but at present its action in this direction is considersd due to its laxative effect upon the upper intestine. The bile simply assists the pancreatic juice in emulsifying fat. The sugar-making function of the liver is true, but not peculiar to this organ alone, as many others, even the placenta, possess this property. The most practical point would be to obtain, if possible, sugar in the inceptive stage of cirrhosis, where its presence would settle the distinction between this disease and carcinoma; the latter giving no evidence of the presence of sugar. Lepine has declared that after the ingestion of sugar in cases of cirrhosis, it may be detected in the urine, because the liver cells do not transform it. This is not the case in any disease which does not destroy the liver cells, or in cancer in which the destruction is for the most part local.

Dr. Forchheimer remarked that the physiological function of the bile is inapplicable to the study of the pathology of the liver on the grounds already expressed. Nevertheless it is to be desired that in those morbid conditions connected with the function of the bile a greater exactness in designating the nature of the disease be made. The word "biliousness," in the sense generally used, is an expression so vague and obscure that it ought to be expunged from the list of medical terms. In the majority of instances it is used to denote gastric or gastro-duodenal catarrh. Why not then call the disease by its right name? The engorgement of the liver, which gives rise to the name "biliousness," is caused by this derangement of the gastro-intestinal canal. In very young children the condition is especially observable.

Dr. Seely replied that, if the function of the liver be not yet exactly determined and the connection between the action of the bile in health and any impairment of this secretion in disease be not clearly understood, we certainly shall gain nothing by dropping the word "biliousness." We have no substitute for it, and consequently should gain nothing by adopting another which is not more expressive of the exact character of biliary derangement. The previous speaker would prescribe for a case of gastric or gastro-intestinal catarrh the same remedies with which we treat "biliousness," therefore, the practical result is the same."

The idea that mercury has action upon the liver may be, and doubtless is, erroneous, but it does not seem at all improbable that the old therapeusis of the so-called bilious state was in advance of its true pathology, and he thought that, clinically, at least, we had better have a correct therapeusis than a correct pathology and a defective therapeuis. He could not but regard the present treatment, if the theory of the difficulty is correct, as inferior to the old. It has been shown that catarrh of the mucous membrane of the eyelids could be readily combatted by a preparation of mercury. He would simply claim that the therapeusis did not tally with the theory.

*Malignant Disease of the Liver.*—Dr. Cleveland also reported a case of malignant growth of the liver that rapidly proved fatal. The man was fifty-seven years old and had always enjoyed good health up to within four months before his death. Four months ago he found himself beginning to grow weaker and rapidly emaciating, his appetite failed him and what he ate produced a sensation of fullness and a feeling of unrest, his bowels were regular, he had occasional attacks of mucous vomiting. Twenty-five days before his death, he saw the patient for the first time, he found him anæmic, emaciated and very feeble, inclined to lie in bed, though able to sit up. He complained of weakness, loss of appetite, epigastric fullness and some pain and eructations of gas, bowels not constipated. There was fullness over the region of the liver. This tumor, very hard and firm to the touch, extended to within two inches of the anterior superior process of ilium, over to the umbilious and across to the left hypochondrium ; the outlines seemed to b3 the borders of an enlarged liver, over the surface of the tumor were several hard lumps, one among them especially had a doughy or gummatous feel. There was no fever at any time ; the cachexia was striking. From the time first seen the patient declined in strength very rapidly and in two months was not able to get out of bed. The tumor seemed to increase rapidly in size from week to week, this may have been more apparent than real, on account of the rapid emaciation. The patient sank rapidly and died of asthena twenty-five days after first seen. Only the history of four months of sickness could be obtained, and the patient was not aware that he had a tumor until the doctor made his first visit. No cancer in the family history. No history of syphilis. The habits of the man were said to have been good. The history of the case was considered as pointing to cancer. It was a matter of regret that no post mortem could be obtained.

Dr. Whitaker remarked it was exceedingly to be regretted that a post mortem examination of the case just mentioned could not have been obtained. According to the symptoms described the case was obscure, the only fact upon which to base a diagnosis being the enlargement of the liver. This might have been only apparent and dependent simply on a depression of the organ by an emphysematous lung or a pleuritic effusion. He would, however, exclude this supposition, from the fact that the reporter of the case certainly would have observed either of these conditions. The enlargement might depend upon various causes, syphilis, amyloid degeneration, or carcinoma. Syphilitic enlargement is not so extensive as that in the case reported, moreover, a specific history facilitates the diagnosis in this affection. Nodules may be due to cirrhosis, echinococcus or cancer. In the first mentioned disease they appear, of course, only in the second stage. Amyloid degeneration is not nodular but uniform. This reduces the diagnosis in this case to echinococcus or cancer. Echinoccus does not grow so rapid as cancer and may extend over many years. The habits of the individual also play an important role in this disease, as it is mostly seen in people of association with dogs. Dogs have a habit of approximating the two ends of the alimentary canal, and thus the hydatids are transmitted from the mouth of the animal to the human mouth. It is also to be regretted that no aspiration was made in the case recorded, as the absence of albumen and the presence of the hooklets of the parasites in the fluid would verify the diagnosis in this disease. A post mortem examination might have revealed also a cancer of the stomach, as cancer of the liver is generally secondary to that of the stomach, and but rarely primary. A cancer of the liver may be latent for a time, but this is not so apt to be the case as when the stomach is thus diseased. The rapidity of the course and the extent of the enlargement, then, would lead the speaker to concur in the opinion of the reporter of the case, *i.e.*, cancer of the liver, probably secondary, dependent on cancer of the stomach.—*Lancet and Clinic.*

